# Inferring Differential Networks by Integrating Gene Expression Data With Additional Knowledge

**DOI:** 10.3389/fgene.2021.760155

**Published:** 2021-11-11

**Authors:** Chen Liu, Dehan Cai, WuCha Zeng, Yun Huang

**Affiliations:** ^1^ Department of Chemotherapy, The First Affiliated Hospital of Fujian Medical University, Fuzhou, China; ^2^ Department of Electrical Engineering, City University of Hong Kong, Hong Kong, China; ^3^ Department of Geriatric Medicine, The First Affiliated Hospital of Fujian Medical University, Fuzhou, China

**Keywords:** single-cell RNA sequencing, differential network analysis, prior information, graphical model, gene regulatory network

## Abstract

Evidences increasingly indicate the involvement of gene network rewiring in disease development and cell differentiation. With the accumulation of high-throughput gene expression data, it is now possible to infer the changes of gene networks between two different states or cell types via computational approaches. However, the distribution diversity of multi-platform gene expression data and the sparseness and high noise rate of single-cell RNA sequencing (scRNA-seq) data raise new challenges for existing differential network estimation methods. Furthermore, most existing methods are purely rely on gene expression data, and ignore the additional information provided by various existing biological knowledge. In this study, to address these challenges, we propose a general framework, named weighted joint sparse penalized D-trace model (WJSDM), to infer differential gene networks by integrating multi-platform gene expression data and multiple prior biological knowledge. Firstly, a non-paranormal graphical model is employed to tackle gene expression data with missing values. Then we propose a weighted group bridge penalty to integrate multi-platform gene expression data and various existing biological knowledge. Experiment results on synthetic data demonstrate the effectiveness of our method in inferring differential networks. We apply our method to the gene expression data of ovarian cancer and the scRNA-seq data of circulating tumor cells of prostate cancer, and infer the differential network associated with platinum resistance of ovarian cancer and anti-androgen resistance of prostate cancer. By analyzing the estimated differential networks, we find some important biological insights about the mechanisms underlying platinum resistance of ovarian cancer and anti-androgen resistance of prostate cancer.

## 1 Introduction

Biological systems often involve the complex regulatory relationships between genes, which could change substantially in different states or developmental stages. Inferring the changes of gene regulatory networks between two different states or cell types is important for revealing the regulatory mechanisms relevant to disease development and cell differentiation (Tian et al., 2016; Zhang et al., 2017). With the accumulation of state-specific gene expression data, a great number of computational approaches have been proposed for estimating gene regulatory networks as well as their difference between two distinct states from gene expression data (Danaher et al., 2014; Ha et al., 2015; Lichtblau et al., 2016; Tian et al., 2016; Zhang et al., 2016; Ou-Yang et al., 2017; Uppal et al., 2018).

Due to the ability in capturing the conditional dependencies among genes, Gaussian graphical models have been widely used to infer gene regulatory networks (Danaher et al., 2014; Zhang et al., 2016; Yuan et al., 2017; Ou-Yang et al., 2019). Existing Gaussian graphical model-based differential network estimation methods can be roughly divided into two categories, i.e., indirect estimation models (Danaher et al., 2014; Zhang et al., 2016) and direct estimation models (Tian et al., 2016; Yuan et al., 2017). Indirect estimation models first estimate each state-specific network separately and then infer the differential network by calculating the difference between two state-specific networks (Danaher et al., 2014). Whereas direct estimation models directly estimate the difference between two state-specific networks without the need to estimate individual state-specific networks (Tian et al., 2016). As the number of parameters that needs to be estimated in direct estimation models is half of that in indirect estimation models, direct estimation models usually achieve better performance than indirect estimation models in differential network estimation, especially in the case of small sample size (Yuan et al., 2017).

Although the above models have been successfully used to infer differential networks (Danaher et al., 2014; Tian et al., 2016; Yuan et al., 2017), they are mainly designed for bulk tissue gene expression data collected from a single data platform. Recently, with the development of high-throughput experimental technologies, we are able to collect bulk gene expression data of same samples from multiple data platforms. As the gene expression data collected from different data platforms may provide some shared and specific information about the regulatory relationships between genes, integrating multi-platform gene expression data could help to improve the accuracy of differential network estimation (Zhang et al., 2016, 2017). Moreover, the advance of single-cell RNA sequencing (scRNA-seq) techniques offers a great opportunity for inferring the regulatory relationships between genes at single cell resolution. The accumulation of scRNA-seq data paves the way to infer cell-type-specific gene networks, which could help to explore the heterogeneity between different cell types (Pratapa et al., 2020). However, due to technical limitations of existing scRNA-seq technologies, a truly expressed gene may not be identified in some cells, which leads to excess of false zeros in scRNA-seq data (i.e., dropout events) (Stegle et al., 2015). Existing differential network estimation models usually assume that the observed data are complete, and rarely consider missing value problem. To handle the distribution diversity of multi-platform gene expression data and the sparseness of single-cell RNA sequencing (scRNA-seq) data, Ou-Yang et al. (2021) proposed an indirect differential network estimation model, which can integrate the gene expression data collected from multiple data platforms and tackle the missing value problem. Moreover, their model can take into account the changes in gene expression levels when inferring differential networks.

The above models only use gene expression data to infer differential networks. However, since the number of samples are usually much smaller than the number of genes, and scRNA-seq data are much sparser and noisier than bulk RNA-seq data, it is difficult to infer differential networks accurately only based on gene expression data. Besides gene expression data, existing knowledge of genes and knowledge of the regulatory relationships among genes may also help to improve the accuracy of differential network estimation (Xu et al., 2018). For example, we can collect some literature-curated gene regulatory interactions from public database (Han et al., 2015). As the changes of regulatory relationships between two different states is more likely to occur between genes that are known to have regulatory interactions, considering prior gene regulatory interactions may help to improve the accuracy of differential network estimation. Moreover, researchers have found that genes within same pathways usually interact with each other to carry out their biological functions, and genes belong to different pathways seldom interact with each other (Wu et al., 2019). Thus, taking into account pathway information may also facilitate the inference of differential networks.

In this study, to address the above problems and provide a differential network estimation method that can generally work well on different types of data, we propose a novel method named Weighted Joint Sparse penalized D-trace Model (WJSDM). Our model can directly estimate the differential networks between two different states by integrating multi-platform gene expression data with additional biological knowledge. Similar to (Ou-Yang et al., 2021), based on non-paranormal graphical model and revised Kendall’s tau correlation, our model can tackle non-Gaussian data with missing values, which make it able to deal with multi-platform gene expression and scRNA-seq data. By using D-trace loss function, our model can estimate the differential network directly, which reduce the number of parameters that need to be estimated. To integrate various prior biological knowledge and take into account changes in gene expression levels, we propose a weighted group bridge penalty. Our model can be solved by using an accelerated proximal gradient method. Simulation studies are first conducted to evaluate the performance of our model. According to the experiment results, our model can always achieve better performance than other state-of-the-art differential network estimation models, which demonstrate the effectiveness of our model in integrating prior information and handling gene expression data with missing values. Extensive experiments on two real data sets also demonstrate the advantages of our model in inferring differential networks and revealing the underlying mechanisms of disease developments. The source code of our proposed model is available at https://github.com/Yunhuang85/WJSDM.

## 2 Methods

In this section, we will first review the non-paranormal distribution and D-trace loss. Then we will introduce our weighted joint sparse penalized D-trace model.

### 2.1 Non-paranormal Distribution

Let *X* = (*X*_1_, *X*_2_, … , *X*_*p*_) denote a *p*-dimensional random vector which follows a multivariate normal distribution *X* ∼ *N*(0, Σ), where $\Sigma \in R^{p\times p}$ is the covariance matrix. For multivariate normal distributions, *X*_*i*_ is independent of *X*_*j*_ given the other variables if and only if the corresponding entry in the inverse covariance matrix (precision matrix) Θ = Σ^−1^ is equal to zero, i.e., Θ_*ij*_ = 0. Thus, the conditional dependence relationships among *p* random variables in *X* can be obtained by identifying the nonzero elements in Θ. However, the normal distribution assumption is too restrictive in practice. To relax the normal distribution assumption, non-paranormal distribution is proposed. *X* = (*X*_1_, *X*_2_, … , *X*_*p*_) is said to follow a non-paranormal distribution *X* ∼ *NPN*(*f*, Σ) if there exists a set of monotone and differentiable functions ${\left\{f_{j}\right\}}_{j=1}^{p}$ such that *f*(*X*) = (*f*_1_(*X*_1_), …, *f*_*p*_(*X*_*p*_)) ∼ *N*(0, Σ). It has been proven that Θ = Σ^−1^ encodes the conditional dependence relationships among *X*. That is, *X*_*i*_ is independent of *X*_*j*_ given the other variables if and only if Θ_*ij*_ = 0.

### 2.2 D-Trace Loss

Given the gene expression data ${\left\{X^{\left(c\right)}\right\}}_{c=1,2}$ of two different states. Each data set $X^{\left(c\right)}\in R^{n_{c}\times p}$ includes *n*_*c*_ samples and *p* common genes. Suppose the *n*_*c*_ samples within each data set are from the same non-paranormal distribution *NPN*(*f* ^(*c*)^, Σ^(*c*)^), where $\Sigma^{\left(c\right)}\in R^{p\times p}$ is the covariance matrix. The conditional dependence relationships between these *p* genes can be inferred from the precision matrix $\Theta^{\left(c\right)}={\left(\Sigma^{\left(c\right)}\right)}^{-1}$ . Thus, the difference between two state-specific networks can be presented as Δ = Θ^(2)^ − Θ^(1)^. To estimate the differential network Δ efficiently, we can utilize the following D-trace loss function (Yuan et al., 2017), which could directly estimate the difference between two precision matrices without separate estimation of each precision matrix:

$$\underset{\Delta =\Delta^{T}}{\arg\;\min}\;L_{D}\left(\Delta ;\Sigma^{\left(1\right)},\Sigma^{\left(2\right)}\right)=\frac{1}{4}\left(\left\langle \Sigma^{\left(1\right)}\Delta ,\Delta \Sigma^{\left(2\right)}\right\rangle +\left\langle \Sigma^{\left(2\right)}\Delta ,\Delta \Sigma^{\left(1\right)}\right\rangle \right)-\left\langle \Delta ,\Sigma^{\left(1\right)}-\Sigma^{\left(2\right)}\right\rangle .$$ (1)

where $\left\langle \left(A,B\right)\right\rangle =tr\left(AB^{T}\right)$ . In practice, we need to use the sample covariance matrices ${\hat{\Sigma }}^{\left(c\right)}$ and minimize $L_{D}\left(\Delta ;{\hat{\Sigma }}^{\left(1\right)},{\hat{\Sigma }}^{\left(2\right)}\right)$ with respect to Δ to calculate the estimator of Δ. For non-paranormal distribution, the sample non-paranormal covariance matrix can be computed *via* rank-based correlation estimator, e.g., Kendall’s tau correlation, without estimating the univariate transformation functions *f* ^(*c*)^.

### 2.3 Notations and Problem Statement

Assuming that there are two different groups of samples. As the gene expression data of same samples can be collected from multiple data platforms, suppose we can observe the expression levels of *p* common genes for these two groups of samples from *K* different data platforms, and the *c*th group contains *n*_*c*_ samples, *c* = 1, 2. Let $X^{\left(kc\right)}\in R^{n_{c}\times p}$ denote the gene expression matrix of the *c*th group collected from *k*th platform, where *n*_*c*_ and *p* denote the number of samples and the number of common genes, respectively. Suppose the *n*_*c*_ samples are from the same non-paranormal distribution *NPN*(*f* ^(*kc*)^, Σ^(*kc*)^), where $\Sigma^{\left(kc\right)}\in R^{p\times p}$ is the covariance matrix. Let ${\left\{\Theta^{\left(kc\right)}\right\}}_{k=1,\ldots ,K}^{c=1,2}$ denote the precision matrices for two groups of samples collected from *K* platforms, where $\Theta^{\left(kc\right)}={\left(\Sigma^{\left(kc\right)}\right)}^{-1}$ . For samples collected from the *k*th platform, the difference between two state-specific networks can be presented as Δ^(*k*)^ = Θ^(*k*2)^ − Θ^(*k*1)^. Our goal is to estimate *K* differential networks ${\left\{\Delta^{\left(k\right)}\right\}}_{k=1,\ldots ,K}$ jointly. For the sake of convenience, we denote ${\left\{X^{\left(kc\right)}\right\}}_{k=1,\ldots ,K}^{c=1,2}$ , ${\left\{\Sigma^{\left(kc\right)}\right\}}_{k=1,\ldots ,K}^{c=1,2}$ , ${\left\{\Theta^{\left(kc\right)}\right\}}_{k=1,\ldots ,K}^{c=1,2}$ and ${\left\{\Delta^{\left(k\right)}\right\}}_{k=1,\ldots ,K}$ as {*X* ^(*kc*)^}, {Σ^(*kc*)^}, {Θ^(*kc*)^} and {Δ^(*k*)^}, respectively.

### 2.4 Weighted Joint Sparse Penalized D-Trace Model

The above D-trace loss is designed to infer the differential network between two different groups of samples from a single data platform, and cannot utilize the common information provided by gene expression data collected from multiple data platforms. Thus, in this study, we extend D-trace loss and develop a weighted joint sparse D-trace model (WJSDM), which can draw support from gene expression data collected from multiple data platforms to estimate the differential network between two different states.

According to the above D-trace loss, the loss function *L*_*KD*_ of *K* data platforms can be given by:

$$L_{KD}\left(\left\{\Delta^{\left(k\right)}\right\}\right)=\frac{1}{4}\overset{K}{\underset{k=1}{\sum }}\left(\left\langle {\hat{\Sigma }}^{\left(k1\right)}\Delta^{\left(k\right)},\Delta^{\left(k\right)}{\hat{\Sigma }}^{\left(k2\right)}\right\rangle +\left\langle {\hat{\Sigma }}^{\left(k2\right)}\Delta^{\left(k\right)},\Delta^{\left(k\right)}{\hat{\Sigma }}^{\left(k1\right)}\right\rangle \right)-\overset{K}{\underset{k=1}{\sum }}\left(\left\langle \Delta^{\left(k\right)},{\hat{\Sigma }}^{\left(k1\right)}-{\hat{\Sigma }}^{\left(k2\right)}\right\rangle \right).$$ (2)

where ${\hat{\Sigma }}^{\left(kc\right)}$ is the sample non-paranormal covariance matrix for *c*th group and *k*th data platform, *k* = 1, … , *K* and *c* = 1, 2. As gene expression data may include some missing values, similar to (Wang et al., 2014; Ou-Yang et al., 2021), we adopt a rank-based correlation, i.e., revised Kendall’s tau correlation, to estimate ${\hat{\Sigma }}^{\left(kc\right)}$ . In particular, let $n_{ij}^{\left(kc\right)}=\underset{1\leq l\leq n_{kc}}{\sum }d_{li}^{\left(kc\right)}d_{lj}^{\left(kc\right)}$ denote the number of samples in the *c*th group and *k*th platform that have nonzero expression values for *i*th and *j*th genes simultaneously, where $d_{lj}^{\left(kc\right)}=1$ if $X_{lj}^{\left(kc\right)}\neq 0$ and $d_{lj}^{\left(kc\right)}=0$ if $X_{lj}^{\left(kc\right)}=0$ . The revised Kendall’s tau correlation between *i*th and *j*th genes are defined as follows:

$${\hat{\tau }}_{ij}^{\left(kc\right)}=\frac{1}{n_{ij}^{\left(kc\right)}\left(n_{ij}^{\left(kc\right)}-1\right)}\underset{l\neq l^{\prime }}{\sum }d_{li}^{\left(kc\right)}d_{l^{\prime }i}^{\left(kc\right)}d_{lj}^{\left(kc\right)}d_{l^{\prime }j}^{\left(kc\right)}sign\left(\left(X_{li}^{\left(kc\right)}-X_{l^{\prime }i}^{\left(kc\right)}\right)\left(X_{lj}^{\left(kc\right)}-X_{l^{\prime }j}^{\left(kc\right)}\right)\right)$$ (3)

As Kendall’s tau correlation are invariant under strictly monotone marginal transformations (Liu et al., 2012), $\Sigma_{ij}^{\left(kc\right)}$ can be estimated by the following definition of ${\hat{\Sigma }}_{ij}^{\left(kc\right)}$

$${\hat{\Sigma }}_{ij}^{\left(kc\right)}=\left\{\begin{array}{cc} \sin\left(\frac{\pi }{2}{\hat{\tau }}_{ij}^{\left(kc\right)}\right),\; & \text{ if }i\neq j,\\ 1,\; & \text{ if }i=j. \end{array}\right.$$ (4)

In this study, each sample non-paranormal covariance matrix ${\hat{\Sigma }}_{ij}^{\left(kc\right)}$ is computed according to the revised Kendall’s tau correlation and transformation function defined in Eqs 3, 4. To ensure ${\hat{\Sigma }}^{\left(kc\right)}$ is positive semidefinite, following Zhang et al. (2021) and Higham (1988), we compute the nearest positive semidefinite matrix *S* ^(*kc*)^ of ${\hat{\Sigma }}^{\left(kc\right)}$ and use it to replace ${\hat{\Sigma }}^{\left(kc\right)}$ .

Note that the differential networks inferred from gene expression data collected from different data platforms may share certain network structures, and the differential networks between two different states may be sparse. Furthermore, differentially expressed genes usually tend to change their regulatory relationships with other genes. Thus, to jointly estimate multiple differential networks and consider the changes in expression levels of individual genes when inferring differential networks, similar to (Ou-Yang et al., 2021), we introduce the following group bridge penalty function:

$$P\left(\left\{\Delta^{\left(k\right)}\right\}\right)=\underset{i,j}{\sum }\sqrt{\overset{K}{\underset{k=1}{\sum }}\tau_{ij}^{\left(k\right)}|\Delta_{ij}^{\left(k\right)}|}.$$ (5)

where $\tau_{ij}^{\left(k\right)}=1-\left(1-r_{i}^{\left(k\right)}\right)\left(1-r_{j}^{\left(k\right)}\right)$ can assign different weights to different pairs of genes, and $r_{i}^{\left(k\right)}\in \left[0,1\right]$ denotes the parameter which measures the differential expression level of *i*th gene, inferred from the *k*th experimental platform. In this study, following Ou-Yang et al. (2021), the *p*-value of Wilcoxon rank-sum test is used to calculate $r_{i}^{\left(k\right)}$ , which can reflect the differential expression level of *i*th gene. With this penalty function, the differential networks {Δ^(*k*)^} inferred from *K* different data platforms may have similar patterns of sparsity and have some shared and specific edges.

Besides gene expression data, there are usually some prior biological knowledge that can help to improve the accuracy of differential network estimation, such as pathway information and prior gene interactions. To incorporate these prior information when inferring differential networks, we extend the above group bridge penalty function to the following weighted group bridge penalty function:

$$P\left(\left\{\Delta^{\left(k\right)}\right\}\right)=\underset{i,j}{\sum }W_{ij}\sqrt{\overset{K}{\underset{k=1}{\sum }}\tau_{ij}^{\left(k\right)}|\Delta_{ij}^{\left(k\right)}|}.$$ (6)

Here, *W* = [*W*_*ij*_] is the weight matrix defined by prior knowledge. In this study, the prior information we used includes pathway information and gene interactions that have been verified from other biological studies. Let *G* ∈ {0,1}^*p*×*p*^ and *F* ∈ {0,1}^*p*×*p*^ denote the prior gene interaction and co-pathway indication matrices, respectively, where *G*_*ij*_ = 1 if the *i*th and *j*th genes are known to have regulatory relationship and *G*_*ij*_ = 0 otherwise, *F*_*ij*_ = 1 if the *i*th and *j*th genes belong to at least one common pathway and *F*_*ij*_ = 0 otherwise. To assign different weights to different pairs of genes, we define *W*_*ij*_ as follows:

$$W_{ij}=\left\{\begin{array}{cc} w_{g},\; & \text{ if }G_{ij}=1,\\ 1,\; & \text{ if }G_{ij}=0\;\;\;\text{and}\;\;\;F_{ij}=1,\\ w_{f},\; & \text{if}G_{ij}=0\;\;\;\text{and}\;\;\;F_{ij}=0. \end{array}\right.$$ (7)

where *w*_*g*_ and *w*_*f*_ are two predefined weight parameters. In reality, the differential edges are more likely to take place between gene pairs that are known to have interactions, and the differential edges are less likely to occur between genes that belong to different pathways. Thus, to assign small penalties to gene pairs that are known to have interactions and large penalties to gene pairs that belong to different pathways, the value of *w*_*g*_ should be less than 1 and the value of *w*_*f*_ should be larger than 1. Following previous studies (Xu et al., 2018), in this study, we fix *w*_*g*_ = 0.3 and *w*_*f*_ = 10.

By combining (2) and (6), the objective function of our Weighted Joint Sparse penalized D-trace Model (WJSDM) is given by:

$$\left\{{\hat{\Delta }}^{\left(k\right)}\right\}=\underset{\left\{\Delta^{\left(k\right)}={\left(\Delta^{\left(k\right)}\right)}^{T}\right\}}{\arg\;\min}\;L_{KD}\left(\left\{\Delta^{\left(k\right)}\right\}\right)+\lambda \underset{i,j}{\sum }W_{ij}\sqrt{\overset{K}{\underset{k=1}{\sum }}\tau_{ij}^{\left(k\right)}|\Delta_{ij}^{\left(k\right)}|}.$$ (8)

where *λ* is a non-negative tuning parameter to control the sparsity levels of the estimated differential networks. We use an iterative approach based on local linear approximation (Zou and Li, 2008) and the accelerated proximal gradient method (Parikh and Boyd, 2014; Xu et al., 2018) to solve problem (Eq. 8).

According to (Yuan et al., 2017), the gradient of the D-trace loss function with respect to Δ takes the following form:

$$▽L_{D}\left(\Delta \right)=\frac{1}{2}\left({\hat{\Sigma }}^{\left(1\right)}\Delta {\hat{\Sigma }}^{\left(2\right)}+{\hat{\Sigma }}^{\left(2\right)}\Delta {\hat{\Sigma }}^{\left(1\right)}\right)-\left({\hat{\Sigma }}^{\left(1\right)}-{\hat{\Sigma }}^{\left(2\right)}\right).$$ (9)

Following the proximal gradient method (Parikh and Boyd, 2014), *L*_*KD*_ can be approximated by the following function:

$$\begin{array}{cc} {\tilde{L}}_{KD}\left(\left\{\Delta^{\left(k\right)}\right\};\left\{{\left({\hat{\Delta }}^{\left(k\right)}\right)}^{\left(t\right)}\right\},\left\{l_{k}\right\}\right) & =\overset{K}{\underset{k=1}{\sum }}\left[L_{D}\left({\left({\hat{\Delta }}^{\left(k\right)}\right)}^{\left(t\right)}\right)+tr\left(▽L_{D}\left({\left({\hat{\Delta }}^{\left(k\right)}\right)}^{\left(t\right)}\right)\left(\Delta^{\left(k\right)}-{\left({\hat{\Delta }}^{\left(k\right)}\right)}^{\left(t\right)}\right)\right)\right.\left.+\frac{1}{2l_{k}}\|\Delta^{\left(k\right)}-{\left({\hat{\Delta }}^{\left(k\right)}\right)}^{\left(t\right)}{\|}_{F}^{2}\right]. \end{array}$$ (10)

where ${\left({\hat{\Delta }}^{\left(k\right)}\right)}^{\left(t\right)}$ is the estimation of Δ^(*k*)^ at *t*th iteration, *l*_*k*_ > 0 and $\|A{\|}_{F}^{2}=\overset{p}{\underset{i,j=1}{\sum }}A_{ij}$ . We rewrite the ${\tilde{L}}_{KD}$ function as:

$${\tilde{L}}_{KD}\left(\left\{\Delta^{\left(k\right)}\right\};\left\{{\left({\hat{\Delta }}^{\left(k\right)}\right)}^{\left(t\right)}\right\},\left\{l_{k}\right\}\right)=\overset{K}{\underset{k=1}{\sum }}\left[\frac{1}{2l_{k}}\|\Delta^{\left(k\right)}-\left({\left({\hat{\Delta }}^{\left(k\right)}\right)}^{\left(t\right)}-l_{k}▽L_{D}\left({\left({\hat{\Delta }}^{\left(k\right)}\right)}^{\left(t\right)}\right)\right){\|}_{F}^{2}+\varphi \left({\left({\hat{\Delta }}^{\left(k\right)}\right)}^{\left(t\right)}\right)\right].$$ (11)

where $\varphi \left({\left({\hat{\Delta }}^{\left(k\right)}\right)}^{\left(t\right)}\right)$ is a function of ${\left({\hat{\Delta }}^{\left(k\right)}\right)}^{\left(t\right)}$ .

Algorithm 1 Complete Algorithm for WJSDM (8)
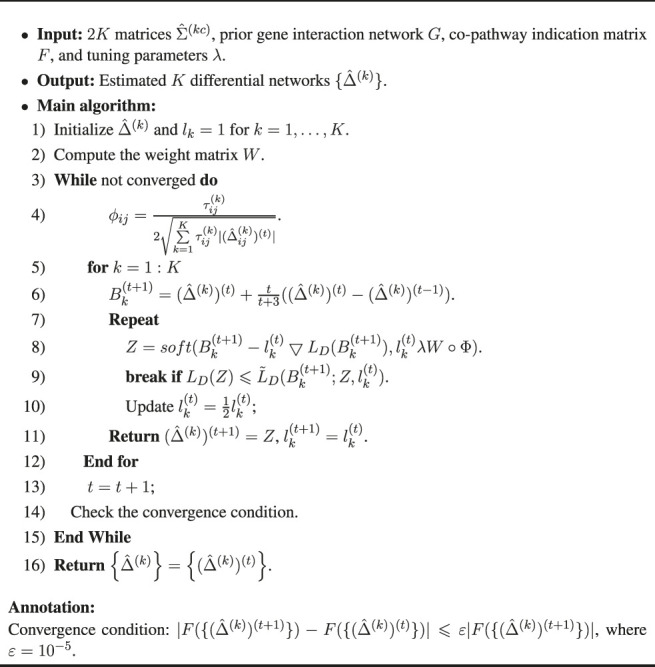

According to local linear approximation (Parikh and Boyd, 2014), Eq. 6 can be approximated as:

$$P\left(\left\{\Delta^{\left(k\right)}\right\}\right)\approx \lambda \overset{K}{\underset{k=1}{\sum }}\underset{i,j}{\sum }\phi_{ij}W_{ij}|\Delta_{ij}^{\left(k\right)}|.$$ (12)

where $\phi_{ij}=\frac{\tau_{ij}^{\left(k\right)}}{2\sqrt{\overset{K}{\underset{k=1}{\sum }}\tau_{ij}^{\left(k\right)}|{\left({\hat{\Delta }}_{ij}^{\left(k\right)}\right)}^{\left(t\right)}|}}$ . Therefore, at (*t* + 1)-th iteration, problem (Eq. 8) can be decomposed into the following *K* individual optimization problems:

$${\left({\hat{\Delta }}^{\left(k\right)}\right)}^{\left(t+1\right)}=\underset{\Delta^{\left(k\right)}={\left(\Delta^{\left(k\right)}\right)}^{T}}{\arg\;\min}\;\frac{1}{2}\|\Delta^{\left(k\right)}-\left(\;{\left({\hat{\Delta }}^{\left(k\right)}\right)}^{\left(t\right)}-l_{k}▽L_{D}\left({\left({\hat{\Delta }}^{\left(k\right)}\right)}^{\left(t\right)}\right)\;\right){\|}_{F}^{2}+\lambda l_{k}\underset{i,j}{\sum }\phi_{ij}W_{ij}|\Delta_{ij}^{\left(k\right)}|.$$ (13)

The solution of our WJSDM is summarized in **Algorithm 1**. The computational complexity of each iteration in **Algorithm 1** is *O*(*Kp* ^3^ + *Kp* ^2^), where *K* is the number of data platforms and *p* is the number of genes.

### 2.5 Parameter Selection

There is a tuning parameter *λ* in WJSDM, which affects the sparsity level of the estimated differential networks. In this study, following previous studies (Zhang et al., 2016), we use a stability approach, named StARS method (Liu et al., 2010; Meinshausen and Bühlmann, 2010), to determine the value of *λ*. The detailed procedure of our parameter selection method is summarized in **Algorithm 2**.

Algorithm 2 Tuning Parameter Selection for WJSDM
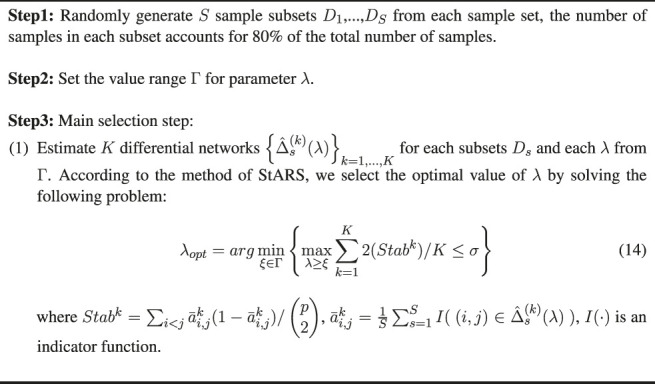

## 3 Results

In this section, we first perform simulation studies to assess the performance of our proposed WJSDM. Then we apply our model on real data sets.

### 3.1 Simulation Studies

To demonstrate the effectiveness of our WJSDM in inferring differential networks, we compare WJSDM with five state-of-the-art differential network estimation models, i.e., FGL (Danaher et al., 2014), TDJGL (Zhang et al., 2016), WDNE (Ou-Yang et al., 2021), GGL (Danaher et al., 2014) and D-trace (Yuan et al., 2017).

#### 3.1.1 Data Generation

In this simulation study, we consider two groups of samples and their observations on *p* common genes collected from *K* = 3 data platforms, and generate six scale-free networks for the two groups of samples and three data platforms. Here, we set *p* = 100 and generate *n*_1_ = *n*_2_ = *n* ∈ {50, 100, 200} observations for each data platform. Each network includes three pathways with 0.4*p* genes per pathway, and there are 0.2*p* genes shared by the second and third pathway. For each pathway, we choose 10% edges as differential edges. To model the heterogeneity between different data platforms, we choose 10% of differential edges to be platform-specific. Since differentially expressed genes tend to change their regulatory relationships with other genes, we select 30% genes as differentially expressed genes and the edges connected to differentially expressed genes are more likely to be differential edges. There are no differential edges between genes belong to different pathways. To make a fair comparison with Gaussian graphical model-based methods, the gene expression levels of each cell are simulated by using a multivariate normal distribution. To generate the prior gene interaction network *G*, we select a prior rate *δ* of nonzero elements from the above six scale-free networks randomly and connect the corresponding genes in *G*. Note that gene expression data may include missing values. In this study, the expression values of a gene may be lost randomly, and the missing rate is set to *τ* ∈ {0.6, 0.8}.

#### 3.1.2 Simulation Results

Let ${\hat{\Delta }}^{\left(k\right)}$ (for indirect estimation methods: ${\hat{\Delta }}^{\left(k\right)}={\hat{\Theta }}^{\left(k1\right)}-{\hat{\Theta }}^{\left(k2\right)}$ ) denote the estimated differential network between two states for the *k*th platform, and Δ^(*k*)^ denote the real differential network for the *k*th platform. We use the following two metrics to evaluate the performance of various algorithms:

$$TPR=\frac{\sum_{k=1}^{K}\sum_{i<j}I\left({\hat{\Delta }}_{ij}^{\left(k\right)}\neq 0\;\text{and}\;\Delta_{ij}^{\left(k\right)}\neq 0\right)}{\sum_{k=1}^{K}\sum_{i<j}I\left(\Delta_{ij}^{\left(k\right)}\neq 0\right)},$$

$$FPR=\frac{\sum_{k=1}^{K}\sum_{i<j}I\left({\hat{\Delta }}_{ij}^{\left(k\right)}\neq 0\;\text{and}\;\Delta_{ij}^{\left(k\right)}=0\right)}{\sum_{k=1}^{K}\sum_{i<j}I\left(\Delta_{ij}^{\left(k\right)}=0\right)}.$$

where *TPR* denotes true positive rate, *FPR* denotes false positive rate, and *I*(⋅) is an indicator function.

**FIGURE 1:**
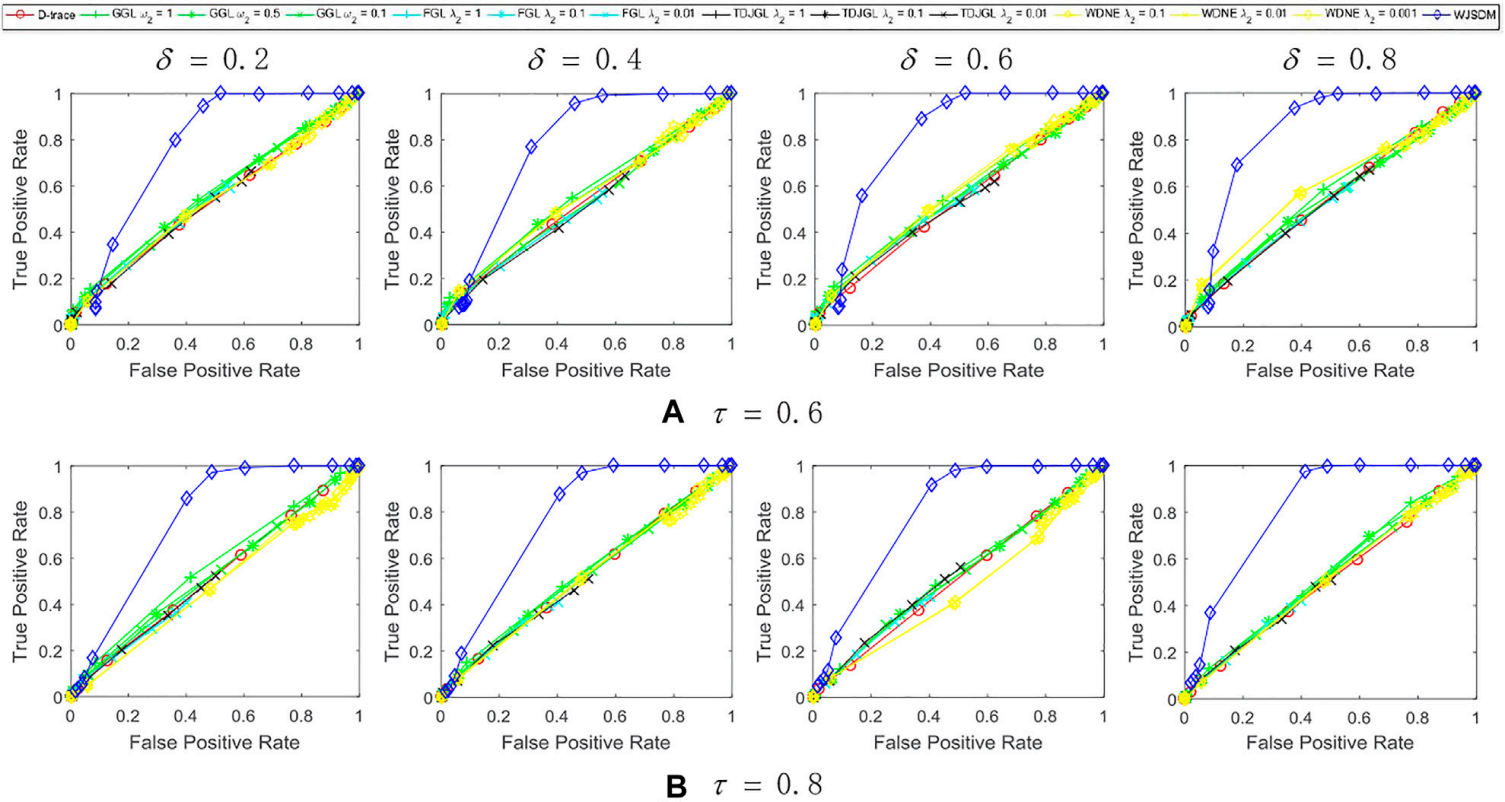
Performance of WJSDM, D-trace, GGL, FGL, TDJGL and WDNE with *p* = 100, *K* = 3, *n* = 50 and missing rate **(A)** τ = 0.6, **(B)** τ = 0.8. Within each plot, each line presents the performance of a method with the value of *λ* (for WJSDM and D-trace), *λ*_1_ (for FGL, TDJGL and WDNE) or *ω*_1_ (for GGL) varying for a fixed value of *λ*_2_ (for FGL, TDJGL and WDNE) or *ω*_2_ (for GGL). Each curve is obtained by averaging the performance of a method over ten random generations of data.

**FIGURE 2:**
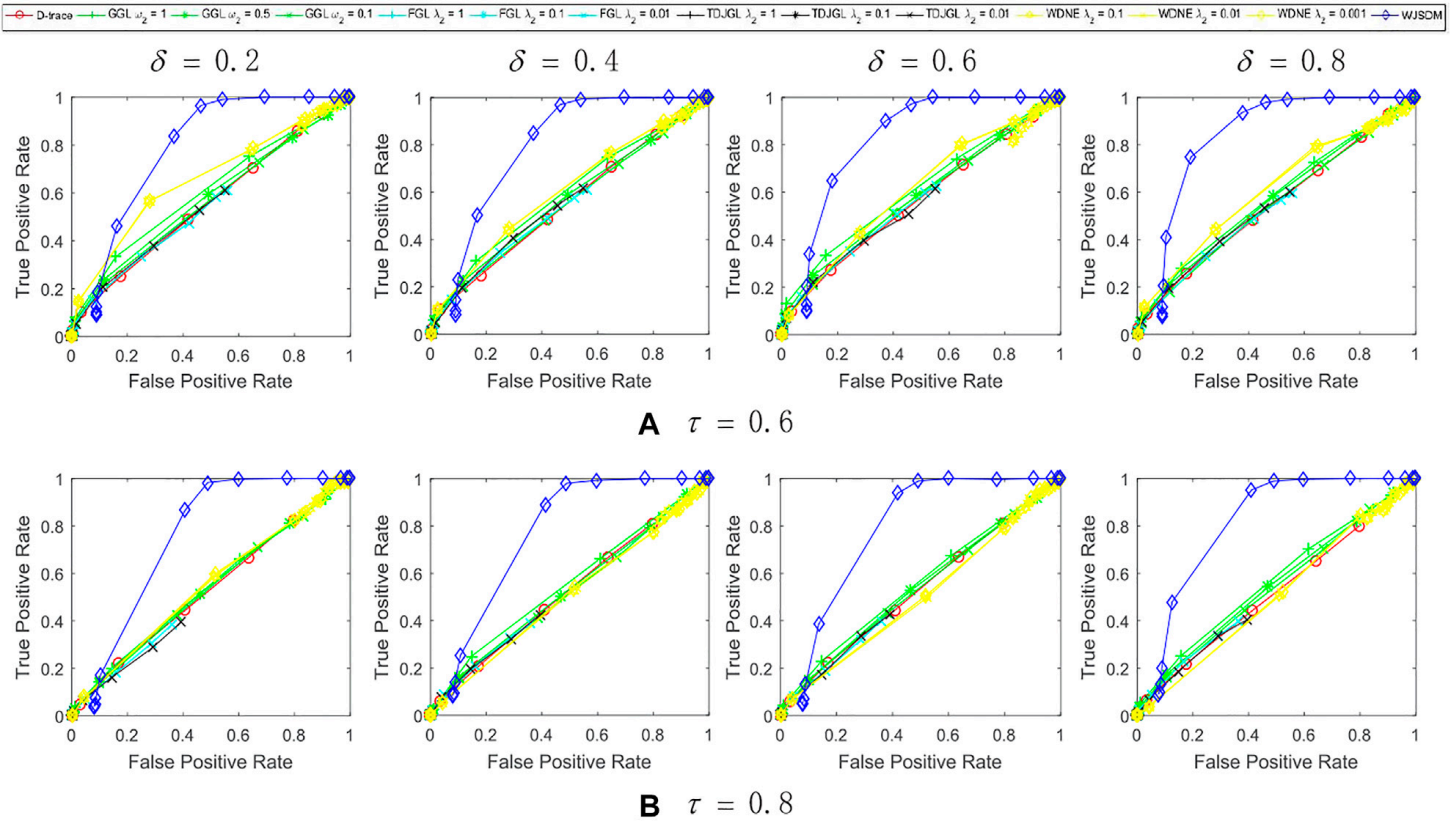
Performance of WJSDM, D-trace, GGL, FGL, TDJGL and WDNE with *p* = 100, *K* = 3, *n* = 100 and missing rate **(A)** τ = 0.6, **(B)** τ = 0.8. Within each plot, each line presents the performance of a method with the value of *λ* (for WJSDM and D-trace), *λ*_1_ (for FGL, TDJGL and WDNE) or *ω*_1_ (for GGL) varying for a fixed value of *λ*_2_ (for FGL, TDJGL and WDNE) or *ω*_2_ (for GGL). Each curve is obtained by averaging the performance of a method over ten random generations of data.

**FIGURE 3:**
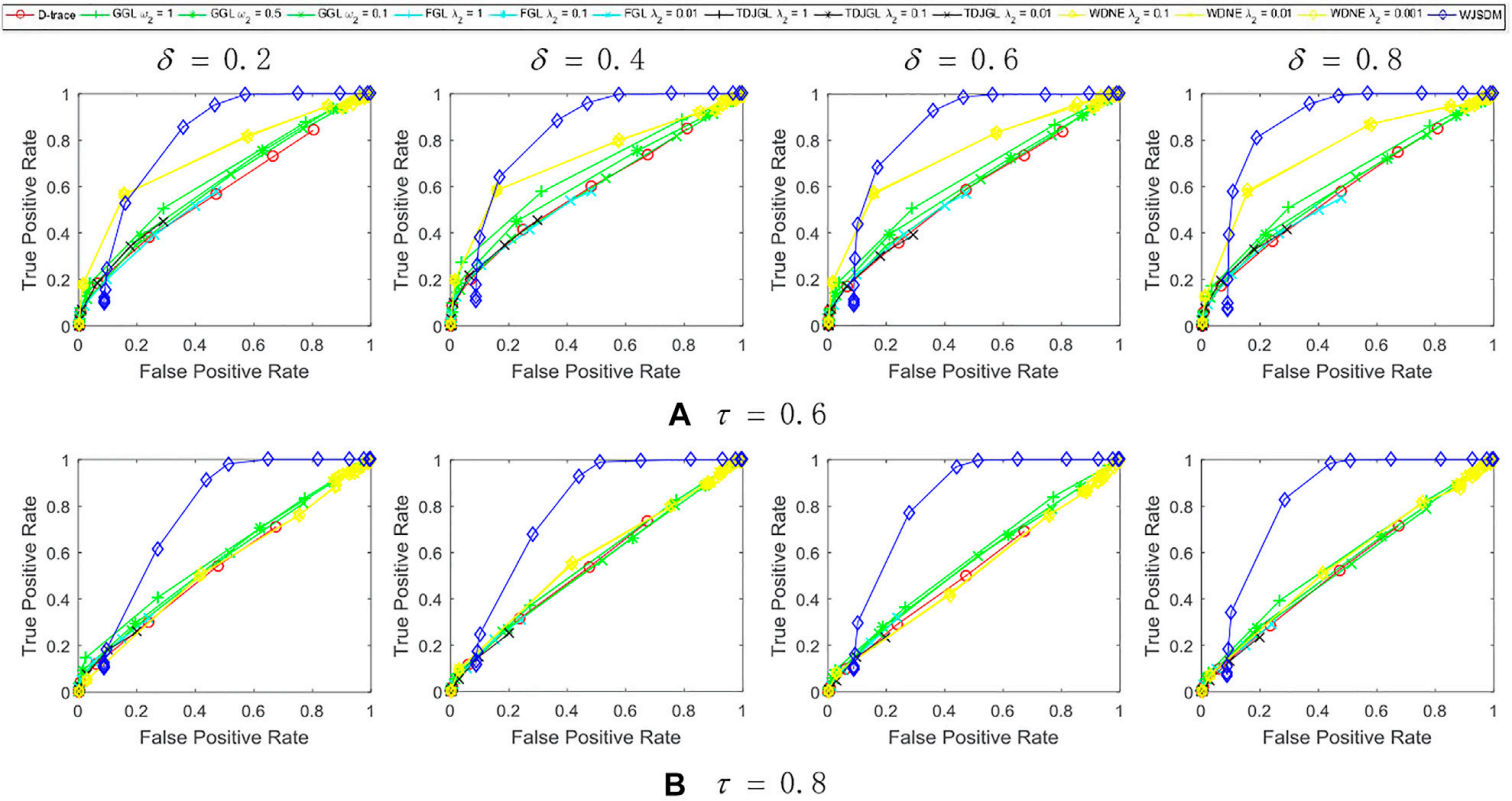
Performance of WJSDM, D-trace, GGL, FGL, TDJGL and WDNE with *p* = 100, *K* = 3, *n* = 200 and missing rate **(A)** τ = 0.6, **(B)** τ = 0.8. Within each plot, each line presents the performance of a method with the value of *λ* (for WJSDM and D-trace), *λ*_1_ (for FGL, TDJGL and WDNE) or *ω*_1_ (for GGL) varying for a fixed value of *λ*_2_ (for FGL, TDJGL and WDNE) or *ω*_2_ (for GGL). Each curve is obtained by averaging the performance of a method over ten random generations of data.

As all methods have some hyper-parameters that need to be predefined, we generate a series of solutions for each model with different values of hyper-parameters, and assess their performances. In particular, for FGL, GGL, TDJGL and WDNE, there are two parameters *λ*_1_ and *λ*_2_. While for D-trace and our WJSDM, there is one parameter *λ*. To ease interpretation, following Danaher et al. (2014), the tuning parameters for GGL are reparameterized as $\omega_{1}=\lambda_{1}+\frac{1}{\sqrt{2}}\lambda_{2}$ and $\omega_{2}=\frac{1}{\sqrt{2}}\lambda_{2}/\left(\lambda_{1}+\frac{1}{\sqrt{2}}\lambda_{2}\right)$ . The experiment results of all methods are averaged over 10 random generations of synthetic data. Figures 1–3 show the performance of various methods on synthetic data. The columns of each figure show the results of various methods with different values of prior rate *δ*, and the rows of this figure show the results with different values of missing rate *τ*. In this figure, each plot shows the *TPR* − *FPR* curves of various methods. Within each plot, different colored lines present the performances of different methods and different points in each line indicate the results with respect to different values of hyper-parameters. The colored lines for D-trace and WJSDM indicate their results as the values of *λ* varied. The colored lines for FGL, GGL, TDJGL and WDNE are obtained by fixing the value of *λ*_2_ (or *ω*_2_ for GGL) and varying the values of *λ*_1_ (or *ω*_1_ for GGL). For *λ*_1_ and *ω*_1_, we choose 15 values ranging from 0.01 to 10 (for WDNE, the value of *λ*_1_ is ranging from 0.01 to 100).

We can find from these figures that our WJSDM outperforms other compared methods in all cases. GGL can estimate multiple networks that share common network structures, but it cannot identify the differences between different networks. FGL and D-trace can infer the changes between different networks, but they cannot integrate the data collected from different data platforms. TDJGL is an extension of FGL, which can integrate multi-platform gene expression data. WDNE is an extension of TDJGL, which can handle gene expression data with missing values and take into account changes in gene expression levels. All of the above methods cannot make use of the prior information provided by additional knowledge when inferring differential networks. WDNE is a indirect differential network estimation model, which need to estimate the state-specific networks in advance. Thus, when the sample size is small, it cannot estimate differential network accurately. As shown in Figure 3, when the sample size is large, WDNE can achieve good performance and outperform other compared methods in most cases. The superior performance of WJSDM over WDNE demonstrates the benefit of inferring differential network directly and integrating multiple additional knowledge.

### 3.2 Real Data Analysis

#### 3.2.1 Ovarian Cancer Analysis

Platinum agents, represented by cisplatin, are the most active cytotoxic drugs in ovarian cancer (Tapia and Diaz-Padilla, 2013). Women with platinum-resistant ovarian cancer continue to have poor survival rates, and effective treatment of platinum resistance still remains the largest unmet need in ovarian cancer (van Zyl et al., 2018). To explore the underlying mechanisms of platinum resistance, we utilize WJSDM to infer the changes of gene regulatory networks between platinum-sensitive and platinum-resistant ovarian tumors. In particular, we collect three gene expression datasets from TCGA database (Network, 2011), which measure gene expression levels from three platforms, i.e., Agilent 244K Custom Gene Expression G450, Affymetrix HT Human Genome U133 Array Plate Set and Affymetrix Human Exon 1.0 ST Array. The expression levels of 8,417 genes for 512 samples are available for all these three platforms. Among the 512 samples, 97 samples are platinum-resistant and 243 samples are platinum-sensitive. Following Zhang et al. (2017), we focus our analysis on seven critical pathways involved in platinum resistance, i.e., apoptosis, cell cycle, ErbB signaling pathway, mismatch repair, nucleotide excision repair, p53 signaling pathway and platinum drug resistance (Kanehisa and Goto, 2000). There are 315 genes in our datasets that belong to these seven pathways. The prior gene interaction network is downloaded from the TRRUST database (Han et al., 2015). There are 361 prior interactions among the 315 genes.

**FIGURE 4:**
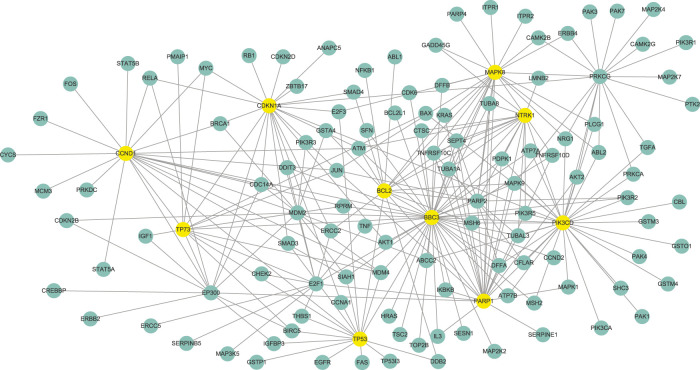
The differential network between platinum-resistant and platinum-sensitive tumors identified by WJSDM. Here, yellow nodes denote the top-10 hub genes in the differential network.

According to the parameter selection strategy (i.e., StARS) introduced above, the tuning parameter *λ* of our WJSDM is set to *λ* = 2.5. The estimated differential network between platinum-resistant and platinum-sensitive tumors, which describes the changes of gene regulatory relationships associated with platinum resistance, is shown in Figure 4. Since we are not able to obtain the true differential network between platinum-resistant and platinum-sensitive tumors, it is hard to measure the accuracy of the estimated differential networks. In fact, a common challenge in evaluating the performance of differential network estimation on real data sets is the lack of reference data. Hub genes in the differential network have more differential edges, which means they may play more important roles in driving the resistance of platinum. Thus, in this study, following previous studies (Zhang et al., 2016, 2017; Ou-Yang et al., 2019), we investigate the functions of the hub genes in our estimated differential network. In particular, the top 10 genes with the highest degree in our estimated differential network are considered as hubs (Table 1). To verify whether our identified hub genes are related to platinum resistance in ovarian cancer, similar to (Zhang et al., 2017), we draw support from six public datasets. In particular, we collect 161 cisplatin resistance-related genes and 758 drug resistance-related genes from the database of Genomic Elements Associated with drug Resistance (GEAR) (Wang et al., 2017), 548 experimentally verified ovarian cancer-related genes from the ovarian cancer gene database (OCGene) (Liu et al., 2015), 116 anti-cancer drug targets from the cancer drug resistance database (CancerDR) (Kumar et al., 2013), 572 cancer genes from the Cancer Gene Census database (Futreal et al., 2004) and 3,545 regulator genes from (Grechkin et al., 2016). Among the identified 10 hub genes, five of them are cisplatin resistance-related genes, eight of them are drug resistance-related genes, six of them are ovarian cancer-related genes, five of them are anti-cancer drug targets, four of them are cancer genes and nine of them are regulator genes.

**TABLE 1:** Top-10 hub genes in the estimated differential gene network between platinum-resistant and platinum-sensitive tumors.

Rank	Gene	Degree	*p*-value	CR	DR	OCG	ADT	CG	RG
1	BBC3	9|38|35	0.023|0.016|0.024						*√*
2	MAPK8	9|21|23	0.306|0.853|0.495	*√*	*√*	*√*	*√*		*√*
3	PIK3CD	7|23|21	0.005|0.006|0.001				*√*		
4	PARP1	7|19|20	0.058|0.028|0.003		*√*	*√*	*√*		*√*
5	CCND1	8|10|21	0.125|0.462|0.071	*√*	*√*	*√*		*√*	*√*
6	TP53	4|16|16	0.702|0.681|0.957	*√*	*√*	*√*		*√*	*√*
7	CDKN1A	4|10|21	0.519|0.557|0.146	*√*	*√*	*√*			*√*
8	TP73	9|11|15	0.073|0.854|0.270		*√*				*√*
9	BCL2	5|13|16	0.592|0.493|0.167	*√*	*√*	*√*	*√*	*√*	*√*
10	NTRK1	11|16|4	0.011|0.098|0.945		*√*		*√*	*√*	*√*

If a gene is a cisplatin resistance-related gene (CR), drug resistance-related gene (DR), ovarian cancer gene (OCG), anti-cancer drug target (ADT), cancer gene (CG) or regulator gene (RG), there is an *√* in the corresponding entry. *a*|*b*|*c* ^*§*^ represents the degree and *p*-values (computed by Wilcoxon rank-sum test) of genes in the differential networks inferred from three platforms, respectively.

Note that the above six public datasets are still far from complete. Thus, we also draw support from literature search to explore whether our identified hub genes are related to cisplatin resistance in ovarian cancer. Among these genes, BBC3 has been reported to be associated with cisplatin resistance in ovarian cancer (Zhang et al., 2012; Grozav et al., 2015), and has been proposed as a chemosensitizer in platinum compounds-based ovarian cancer therapy (Yuan et al., 2011). PARP1 have been shown to involved in cisplatin resistance in ovarian cancer, and could be treated as a potential sensitizer in cisplatin chemotherapy (Liu et al., 2018). TP73 has been found to be associated with clinical responsiveness to platinum-based chemotherapy in advanced non-small cell lung cancer (NSCLC) (Yuan et al., 2006). Researches have found that TP73 could be a genetic marker for ovarian response (Bakay et al., 2021). Thus, it is interesting to study the association between TP73 and platinum resistance in ovarian cancer.

We can also find from Table 1 that our identified hub genes include both differentially (in this study, genes whose *p*-values are less than 0.05 are treated as differentially expressed genes) and non-differentially expressed genes. For example, MAPK8, CCND1, TP53, CDKN1A and BCL2 are related to cisplatin resistance in ovarian cancers. None of these five genes showed differential expression between platinum-resistant and platinum-sensitive tumors. Thus, our model can identify functional important genes that cannot be found by differential expression analysis, which demonstrate the superiority of our model over differential expression analysis.

#### 3.2.2 Prostate Cancer Analysis

Enzalutamide is a second-generation anti-androgen medication which has been used in the treatment of prostate cancer (Scher et al., 2012). However, the mechanisms underlying the resistance of enzalutamide remain vague. We then apply WJSDM to the scRNA-seq data of circulating tumor cells of prostate cancer, and investigate the changes of gene regulatory relationships that associated with enzalutamide-resistant. In particular, we collect a scRNA-seq data set of prostate circulating tumor cells from GEO database with accession number: GSE67980 (Miyamoto et al., 2015). There are 77 samples isolated from 13 patients, where 41 samples are enzalutamide-naive and 36 samples are enzalutamide-resistant (Chiu et al., 2018). Among 21,696 genes, 7,508 genes have no sequencing reads in all the 77 samples. We focus our analysis on three critical pathways download from the Kyoto Encyclopedia of Genes and Genomes database (Kanehisa and Goto, 2000), i.e., Notch signaling pathway, Wnt signaling pathway and PI3K-AKT signaling pathway. By removing genes with no sequencing reads, there are 234 genes in the scRNA-seq data that belong to these three pathways. The prior gene interaction network is downloaded from the TRRUST database (Han et al., 2015). There are 178 prior interactions among the 234 genes.

**FIGURE 5:**
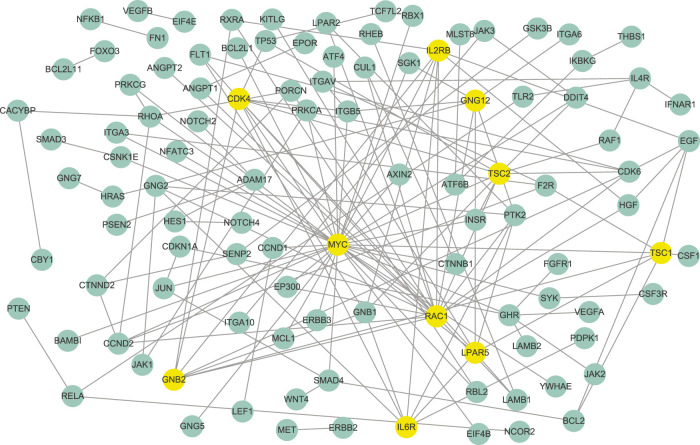
The differential network between enzalutamide-resistant and enzalutamide-naive samples identified by WJSDM. Here, yellow nodes denote the top-10 hub genes in the differential network.

According to the parameter selection strategy (i.e., StARS) introduced above, the tuning parameter *λ* of our WJSDM is set to *λ* = 0.7197. The estimated differential network between enzalutamide-resistant and enzalutamide-naive samples, which describes the changes of gene regulatory relationships associated with enzalutamide resistance, is shown in Figure 5. Hub genes in the differential network have more differential edges, which means they may play more important roles in driving the resistance of enzalutamide. Thus, we investigate the functions of the hub genes in our estimated differential network. In particular, the top 10 genes with the highest degree in our estimated differential network are considered as hubs (as shown in Table 2). We can find from Table 2 that all of these 10 hub genes are related to prostate cancer and five of them are associated with enzalutamide-resistant.

**TABLE 2:** Top-10 hub genes in the estimated differential gene network between enzalutamide-resistant and enzalutamide-naive samples.

Rank	Gene	Degree	*p*-value	PCa	ER
1	MYC	35	0.15	○	○
2	RAC1	18	5.60e-5	○	○
3	CDK4	10	7.84e-4	○	○
4	TSC2	9	0.005	○	○
5	IL2RB	8	0.005	○	
6	LPAR5	8	0.027	○	
7	GNB2	7	0.010	○	
8	GNG12	7	0.024	○	
9	IL6R	7	0.031	○	○
10	TSC1	7	0.010	○	

If the gene is associated with prostate cancer (PCa) or enzalutamide-resistant (ER) according to literature search (Wu et al., 2006; Tam et al., 2007; Lin et al., 2015; Wang et al., 2018; Handle et al., 2019; Chen et al., 2020; Kase et al., 2020; Balijepalli et al., 2021; Dickson et al., 2021; Furlan et al., 2021), a ○ is placed in the corresponding entry. The *p*-value of each gene is computed by Wilcoxon rank-sum test.

Among these genes, MYC has been reported to be implicated in the development of enzalutamide resistance and the increase of MYC expression is correlated with shorter progression free survival in patients undergoing enzalutamide treatment (Handle et al., 2019; Furlan et al., 2021). However, this gene does not show differential expression between enzalutamide-resistant and enzalutamide-naive samples. Thus, it cannot be found by differential expression analysis. RAC1, which has been demonstrated to be upregulated in enzalutamide-resistant prostate cancer cells, plays a crucial role in enzalutamide resistance and could be a potential target for the treatment of castration-resistant prostate cancer (Chen et al., 2020). Knockdown of TSC1 and TSC2 have been shown to promote the proliferation of prostate cancer cells Lin et al. (2015). LPAR5 has been reported to be involved with immune response inhibition and cancer progression Geraldo et al. (2021). Researches have found that GRB2 is associated with shorter survival of patients with aggressive prostate cancer (Iwata et al., 2021). The activation of the IL-6R/JAK/STAT3 pathway has been found to be involved with the development of hormonerefractory prostate cancer (Tam et al., 2007). The combined inhibition of IL6R and HMGB1 has been reported to be a new treatment for enzalutamide resistance in patients with advanced prostate cancer (Wang et al., 2018).

The above results demonstrate the effectiveness of our WJSDM in inferring the difference between the gene networks of different disease states, and provide important insights about the underlying regulatory mechanisms of the platinum resistance in ovarian cancer and the enzalutamide resistance in prostate cancer.

## 4 Conclusion

Increasing evidences indicate the changes of gene regulatory relationships between different cell states or developmental stages, which motivate the development of computational models to infer differential networks. In this paper, based on gene expression data and additional biological knowledge, we propose a novel differential network estimation method named weighted joint sparse penalized D-trace model (WJSDM), to infer the changes of gene regulatory networks between two different states. By employing D-trace loss function and using a revised Kendall’s tau correlation, our method can directly infer the differential network between two different states from gene expression data with missing values. Furthermore, to integrate the gene expression data collected from different data platforms and utilize the information provided by various prior biological knowledge, we propose a weighted group bridge penalty function, which enable our model to draw support from multiple related data sets. Experiment results on synthetic data sets show that compared with other state-of-the-art differential network estimation methods, our method can infer differential networks more accurately. We also apply our method to the gene expression data of ovarian tumors and circulating tumor cells of prostate cancer, and estimate the differential network associated with platinum resistance of ovarian cancer and anti-androgen resistance of prostate cancer. By analyzing our estimated differential networks, we find some important biological insights about the mechanisms underlying platinum resistance of ovarian cancer and anti-androgen resistance of prostate cancer.

With the development of single-cell sequencing techniques, an increasing number of single-cell multi-omics data are becoming available. How to efficiently integrate single-cell multi-omics data is an interesting future work. We will try to extend our model to handle this problem.

## Data Availability

Publicly available datasets were analyzed in this study. This data can be found here: https://portal.gdc.cancer.gov/ https://www.ncbi.nlm.nih.gov/geo/ https://www.kegg.jp/ https://www.grnpedia.org/trrust/.
